# Zinc regulates a key transcriptional pathway for epileptogenesis via metal-regulatory transcription factor 1

**DOI:** 10.1038/ncomms9688

**Published:** 2015-10-26

**Authors:** Karen M. J. van Loo, Christina Schaub, Julika Pitsch, Rebecca Kulbida, Thoralf Opitz, Dana Ekstein, Adam Dalal, Horst Urbach, Heinz Beck, Yoel Yaari, Susanne Schoch, Albert J. Becker

**Affiliations:** 1 Section for Translational Epilepsy Research, Department of Neuropathology, University of Bonn Medical Center, Bonn 53105, Germany; 2 Laboratory for Experimental Epileptology and Cognition Research, Department of Epileptology, University of Bonn Medical Center, Bonn 53105, Germany; 3 Department of Neurology, University of Bonn Medical Center, Bonn 53105, Germany; 4 Department of Medical Neurobiology, IMRIC, Hebrew University–Hadassah School of Medicine, Jerusalem 91120, Israel; 5 Department of Neurology, Hadassah—Hebrew University Medical Center, Jerusalem 91120, Israel; 6 Department of Neuroradiology, Medical Center University of Freiburg, Freiburg 79106, Germany

## Abstract

Temporal lobe epilepsy (TLE) is the most common focal seizure disorder in adults. In many patients, transient brain insults, including status epilepticus (SE), are followed by a latent period of epileptogenesis, preceding the emergence of clinical seizures. In experimental animals, transcriptional upregulation of Ca_V_3.2 T-type Ca^2+^-channels, resulting in an increased propensity for burst discharges of hippocampal neurons, is an important trigger for epileptogenesis. Here we provide evidence that the metal-regulatory transcription factor 1 (MTF1) mediates the increase of Ca_V_3.2 mRNA and intrinsic excitability consequent to a rise in intracellular Zn^2+^ that is associated with SE. Adeno-associated viral (rAAV) transfer of MTF1 into murine hippocampi leads to increased Ca_V_3.2 mRNA. Conversely, rAAV-mediated expression of a dominant-negative MTF1 abolishes SE-induced Ca_V_3.2 mRNA upregulation and attenuates epileptogenesis. Finally, data from resected human hippocampi surgically treated for pharmacoresistant TLE support the Zn^2+^-MTF1-Ca_V_3.2 cascade, thus providing new vistas for preventing and treating TLE.

## Abstract (web summary)

Temporal lobe epilepsy can cause ionic imbalance in the brain and alter transcriptional activities. Here, van Loo *et al.* show that the increase in neuronal zinc following status epilepticus can induce transcriptional change via metal-regulatory transcription factor 1, and alter voltage-gated calcium channel CaV3.2 and intrinsic neuronal excitability.

## Graphical abstract

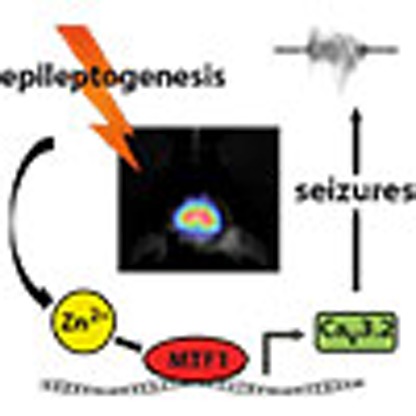

Epilepsy affects 1% of individuals of all ages, socioeconomic backgrounds and races, and is the second most common cause of mental disability, particularly among young adults, accounting for a worldwide disease burden similar to that of breast cancer in women and lung cancer in men^1^,^2^,^3^. Chronic recurrent seizures often originate in the temporal lobe (temporal lobe epilepsy (TLE)) and are pharmacoresistant in approximately a third of the patients. Surgical removal of the epileptic focus, albeit highly effective, represents a therapy option for only a fraction of patients^4^. Often, TLE develops as a consequence of a brain disease or of an acute brain insult (acquired or ‘symptomatic' TLE) via a multifaceted process referred to as epileptogenesis^5^. Intriguingly, a single episode of status epilepticus (SE) can induce the structural and functional alterations that lead to the emergence of chronic recurrent seizures. Identifying the key epileptogenic mechanisms is essential for devising new treatments to prevent or attenuate the development of TLE.

Recent experimental and clinical evidence suggests that acquired ‘transcriptional channelopathies' play a role in epileptogenesis, as well as in the pathogenesis of other neurological disorders^6^,^7^,^8^,^9^. Thus, in rodents, SE induced chemically with pilocarpine (pilocarpine-SE) causes a marked increase in the propensity for intrinsic bursting in hippocampal CA1 pyramidal cells, particularly in the early phase of epileptogenesis^10^. Pharmacological analyses with subtype-selective blockers of voltage-dependent Ca^2+^ channels (VDCCs) disclosed the involvement of a Ni^2+^-sensitive-T-type Ca^2+^ current (*I*_CaT_) in this aberrant activity^11^,^12^. Congruently, *I*_CaT_ was threefold upregulated early after SE, whereas other Ca^2+^ currents were unchanged or even reduced^12^. Furthermore, a significant upregulation of Ca_V_3.2 mRNA, but not that of other T-type Ca^2+^-channel α_1_ mRNAs, was present in SE-experienced neurons and translated to an increase in Ca_V_3.2 protein level^13^. Interestingly, the emergence of epileptic seizures was strongly attenuated in *Ca*_*V*_*3.2* knockout mice subjected to pilocarpine-SE^13^, indicating that transcriptional Ca_V_3.2 upregulation may play a pivotal role in epileptogenesis.

It has been shown that SE causes a rise in intracellular free Zn^2+^ concentrations ([Zn^2+^]_i_) in pyramidal cells^14^ and we have recently demonstrated that Zn^2+^, although acutely and reversibly blocking T-type channels^15^, induces a long-term upregulation of *I*_CaT_ in hippocampal pyramidal cells *in vivo*^16^. Therefore, we have aimed at delineating the signalling cascade linking the increase in [Zn^2+^]_i_ to *Ca*_*V*_*3.2* promoter activation. Our results *in vitro* indicate that this link is mediated by metal-regulatory transcription factor 1 (MTF1). Supporting data obtained from ‘viral transgenic' mice and human TLE hippocampi suggest that MTF1 may be a target for treatment strategies aimed at impeding the development of epilepsy following acute brain insults.

## Results

### Selective induction of Ca_V_3.2 expression by Zn^2+^

**Figure 1:**
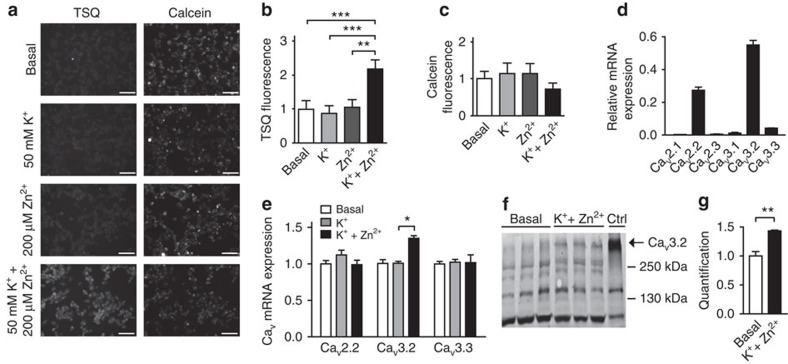
Zn^2+^ increases mRNA and protein expression levels of the T-type Ca^2+^-channel Ca_V_3.2. (**a**) Representative images of the membrane-permeant fluorescent Zn^2+^ indicator TSQ (left row) and calcein red-orange AM fluorescence (right row) in control NG108-15 cultures (basal) and after stimulation with K^+^ (50 mM), Zn^2+^ (200 μM) or K^+^+Zn^2+^ (50 mM/200 μM). Scale bar, 100 μm. (**b**) Quantification of TSQ and (**c**) calcein red-orange fluorescence after incubation with K^+^, Zn^2+^ or K^+^+Zn^2+^ normalized to control cultures (basal). Fluorescence intensity was quantified, background corrected for single cells and then averaged for every field of view. Normalization was performed for simultaneously treated and measured cultures (*n*=5 in two independent experiments; **P≤0.01; ***P≤0.001; repeated measures analysis of variance (ANOVA), multiple Bonferroni's multiple comparison test). (**d**) Relative mRNA expression of the voltage-dependent calcium channels in NG108-15 cells. Only Ca_V_2.2, Ca_V_3.2 and Ca_V_3.3 are expressed above background levels. (**e**) Quantitative RT–PCR for Ca_V_2.2, Ca_V_3.2 and Ca_V_3.3 mRNA levels in NG108-15 cells stimulated with K^+^ or K^+^+Zn^2+^. mRNA expression was measured 4 h after stimulation, with synaptophysin as reference gene (one-way ANOVA: *P*=0.0142; *F*_(8,27)_=3.045; Tukey's multiple comparisons test, *P≤0.05; *n*=4). (**f**) Immunoblot for the ∼260 kDa Ca_V_3.2 protein in NG108-15 cells 3 days after incubation basal or K^+^+Zn^2+^ solutions (50 mM/200 μM). As a control, HEK-293 cells stably expressing Ca_V_3.2 were probed. (**g**) Quantification based on α-tubulin levels (*t*-test: **P≤0.01; *n*=3).

To unravel the signalling cascades underlying Zn^2+^-induced *I*_CaT_ upregulation, we first examined the effect of elevating intracellular Zn^2+^ ([Zn^2+^]_i_) on VDCC gene expression in neural NG108-15 cells. A large increase in [Zn^2+^]_i_ was triggered by incubating the cells with a solution containing 200 μM Zn^2+^ (a concentration that can be reached in pathological conditions such as ischemia, seizures and brain trauma^17^) and high K^+^ (50 mM to depolarize the cells^18^,^19^). Only this combination (further referred to as K^+^+Zn^2+^), but not with K^+^ or Zn^2+^ solutions alone, caused an increase in [Zn^2+^]_i_ (Fig. 1a,b). None of these treatments affected cell viability (Fig. 1c).

Next, we analysed the mRNA expression levels of the neuronal L-type (Ca_V_1.2 and Ca_V_1.3), P/Q-type (Ca_V_2.1), N-type (Ca_V_2.2), R-type (Ca_V_2.3) and T-type (Ca_V_3.1, Ca_V_3.2 and Ca_V_3.3) VDCCs in NG108-15 cells incubated with basal, K^+^ or K^+^+Zn^2+^ solutions. In basal condition, high expression levels were obtained for Ca_V_3.2 and to a lesser degree for Ca_V_2.2 and Ca_V_3.3 (Fig. 1d). Incubation with K^+^+Zn^2+^ solution resulted in a significant upregulation of Ca_V_3.2 mRNA levels, but did not affect mRNA levels of other VDCCs that are sensitive to block by submolar concentrations of Ni^2+^ (Fig. 1e, Supplementary Fig. 1). Furthermore, incubating NG108-15 cells with K^+^ solutions containing other divalent cations (0.5 mM Ni^2+^ or 1 mM Cu^2+^) did not affect Ca_V_3.2 mRNA expression (Supplementary Fig. 2). Thus, a rise in [Zn^2+^]_i_ uniquely and selectively enhances Ca_V_3.2 mRNA expression, an effect that is expected to cause an increase in Ca_V_3.2 protein level. Indeed, immunoblotting detected the Ca_V_3.2 protein under basal conditions and revealed a significant increase of Ca_V_3.2 protein levels after exposure of the cells to K^+^+Zn^2+^ solution (Fig. 1f,g).

### Increase of *I*_CaT_ by Zn^2+^

**Figure 2:**
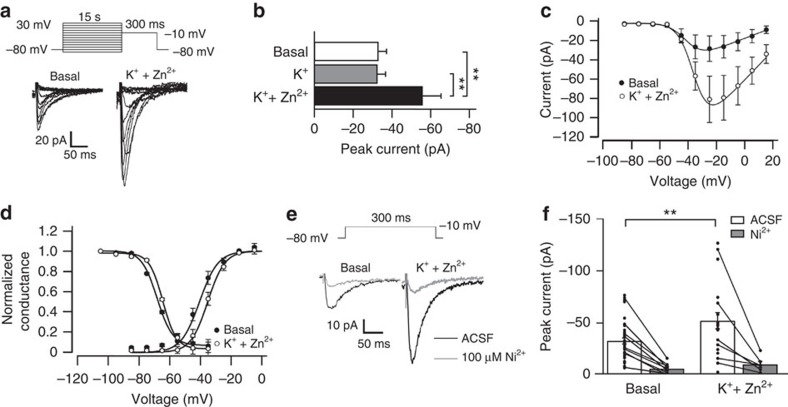
Increase of the T-type current by Zn^2+^. (**a**) Analysis of *I*_CaT_ of NG108-15 cells stimulated with K^+^+Zn^2+^ (50 mM/200 μM) for 4 h, measured 1 day after stimulation. Ca^2+^ currents were recorded in the presence of Na^+^ and K^+^ current blockers (tetraethylammonium 20 mM, 4-AP 4 mM and tetrodotoxin 1 μM). Cells were depolarized from a holding potential of −80 mV to various potentials ranging from −90 to +30 mV, yielding transient, predominantly T-type Ca^2+^ currents. Representative current families elicited with the voltage protocol (upper panel) under basal conditions (left) and following increases in [Zn^2+^]_i_. (**b**) Quantification of the peak *I*_CaT_ elicited by a step to −10 mV under the different conditions; basal: *n*=37 cells; K^+^: *n*=11 cells; K^+^+Zn^2+^: *n*=22 cells; *t*-test: **P≤0.01). (**c**) Current–voltage relationship of T-type currents under basal conditions and following incubation with K^+^+Zn^2+^ (filled circles basal, *n*=3; open circles K^+^+Zn^2+^, *n*=3). (**d**) The voltage dependence of *I*_CaT_ activation and inactivation was unaltered in cells treated with K^+^+Zn^2+^ compared with untreated controls (filled circles basal, activation *n*=3, inactivation *n*=9; open circles K^+^+Zn^2+^, activation *n*=3, inactivation *n*=6). (**e**) Ca^2+^ currents were elicited with a voltage step from −80 to −10 mV (upper part). Representative current traces show an increased amplitude in cells incubated with K^+^+Zn^2+^ (lower trace right) compared with cells under basal conditions (lower trace left). Representative current traces showed the potent block by 100 μM Ni^2+^, indicating involvement of Ca_V_3.2 channels (grey trace). (**f**) Average of the transient Ca^2+^ current of all cells for control cells (basal, *n*=37) and cells incubated with K^+^+Zn^2+^ (*n*=22) display *I*_CaT_ upregulation following incubation with K^+^+Zn^2+^ (*t*-test: **P≤0.01). Average of the transient currents after application of 100 μM Ni^2+^ showed a large amplitude reduction in all recorded cells (−33.1±3.0 pA, *n*=37 versus −6.5±1.7 pA, *n*=9 for basal conditions and −52.2±7.8 pA, *n*=22 versus −10.4±3.0 pA, *n*=6 following incubation with K^+^+Zn^2+^).

We next used whole-cell patch-clamp recordings of Ca^2+^ currents in NG108-15 cells to investigate whether the Zn^2+^-induced increase in Ca_V_3.2 protein is reflected functionally in a larger *I*_CaT_. NG108-15 cells were incubated in either K^+^ or K^+^+Zn^2+^ solutions and Ca^2+^ currents were recorded. Representative examples of *I*_CaT_ in a control cell and in a cell exposed to K^+^+Zn^2+^ solution are shown in Fig. 2a. We found that exposing the cells to K^+^+Zn^2+^ solution, but not to K^+^ solution led to a significant increase in *I*_CaT_ (Fig. 2b,c). This increase was not accompanied by a change in voltage dependence of activation or inactivation (Fig. 2d, Supplementary Table 1). Expectedly, application of 100 μM Ni^2+^, a dose potently inhibiting Ca_V_3.2 channels (grey traces in Fig. 2e), almost completely reduced *I*_CaT_ in both basal and K^+^+Zn^2+^ conditions (Fig. 2f). Altogether, these findings are congruent with the notion that Zn^2+^-induced increase in *I*_CaT_ reflects an increase in functional Ca_V_3.2 channels.

### Zn^2+^-induced activation of the *Ca*_*V*_ *3.2* promoter

**Figure 3:**
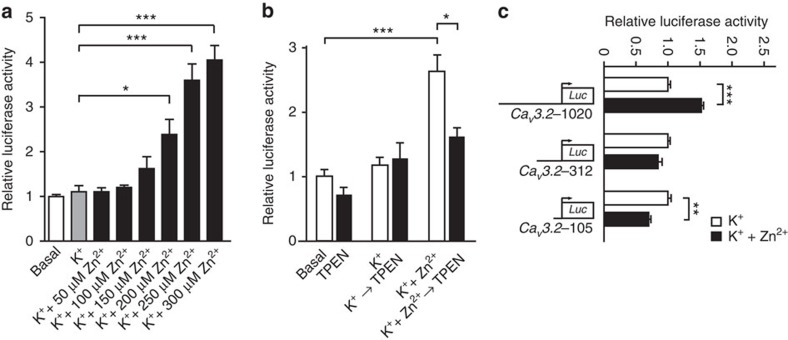
Increases in [Zn^2+^]_i_ activate the *Ca*_*V*_*3.2* promoter. (**a**) NG108-15 cells transfected with the 1,188-bp *Ca*_*V*_*3.2* promoter–luciferase reporter construct^20^ and stimulated with K^+^+Zn^2+^ in the presence of increasing Zn^2+^ concentrations (0, 50, 100, 150, 200, 250 and 300 μM). The effect on the promoter activity was determined using a luciferase assay 4 h after stimulation (one-way analysis of variance (ANOVA): *P*<0.001; *F*_(7,16)_=25.64; Tukey's multiple comparisons test, *P≤0.05, ***P≤0.001; *n*=3). (**b**) Activity of the *Ca*_*V*_*3.2* promoter–luciferase reporter gene determined after stimulation of NG108-15 cells first with K^+^ or K^+^+Zn^2+^ (50 mM/500 μM) solutions for 1 h and subsequently incubated in the presence or absence of TPEN (10 μM) for 1 h. Luciferase activity was measured 4 h after stimulation (one-way ANOVA: *P*<0.001; *F*_(5,10)_=24.63; Tukey's multiple comparisons test,*P≤0.05, ***P≤0.001; *n*≥3). (**c**) Luciferase activity of three *Ca*_*V*_*3.2* promoter deletion fragments^20^ after stimulation with K^+^+Zn^2+^ (50 mM/200 μM). Only the *Ca*_*V*_*3.2*-1020 deletion fragment showed significant activation of the *Ca*_*V*_*3.2* promoter after stimulation with K^+^+Zn^2+^. The short *Ca*_*V*_*3.2*-105 showed a significantly reduced activity after stimulation with K^+^+Zn^2+^, likely due to the presence of Zn^2+^-inhibitory regions within this fragment (one-way ANOVA: *P*<0.001; *F*_(5,12)_=39.82; Tukey's multiple comparisons test, **P≤0.01,***P≤0.001; *n*=3).

We next sought to identify the molecular mechanisms underlying the Zn^2+^-induced upregulation of Ca_V_3.2. To that end, we made use of the previously identified *Ca*_*V*_*3.2* promoter^20^, the activity of which strongly correlates with endogenous Ca_V_3.2 mRNA levels. We found that treating the cells with K^+^+Zn^2+^ solution, but not with K^+^ solution, resulted in a significant *Ca*_*V*_*3.2* promoter activation (Fig. 3a). This effect was enhanced by raising Zn^2+^ concentration above 200 μM. We further examined whether Zn^2+^-induced activation of the *Ca*_*V*_*3.2* promoter reverses on Zn^2+^ removal. To that end, we exposed cells having been incubated in a K^+^+Zn^2+^ solution to N,N,N′,N′-tetrakis(2-pyridylmethyl)ethane-1,2-diamine (TPEN), a cell permeable Zn^2+^ chelator. Intriguingly, TPEN significantly reduced *Ca*_*V*_*3.2* promoter activity. In contrast, basal activity of the *Ca*_*V*_*3.2* promoter was unaffected by TPEN (Fig. 3b). These data suggest that enhanced activation of the *Ca*_*V*_*3.2* promoter requires a sustained increase in [Zn^2+^]_i_.

We next delineated the region responsible for Zn^2+^-induced *Ca*_*V*_*3.2* promoter activation by analysing *Ca*_*V*_*3.2* promoter deletion luciferase reporter constructs (*Ca*_*V*_*3.2*-1020, -312 and -105) (ref. 20). Increases in [Zn^2+^]_i_ resulted in a significant *Ca*_*V*_*3.2* promoter activity only for the longest deletion fragment (Fig. 3c) suggesting that the Zn^2+^-responsive regulatory element is located in the genomic between 312 and 1,020 upstream of the *Ca*_*V*_*3.2* ATG. These findings indicate that *Ca*_*V*_*3.2* gene regulation is under control of a Zn^2+^-responsive transcription factor^21^. The only currently known transcription factor fulfilling these criteria in mammals is MTF1.

### MTF1 increases *Ca*_*V*_ *3.2* promoter activation and *I*_CaT_

**Figure 4:**
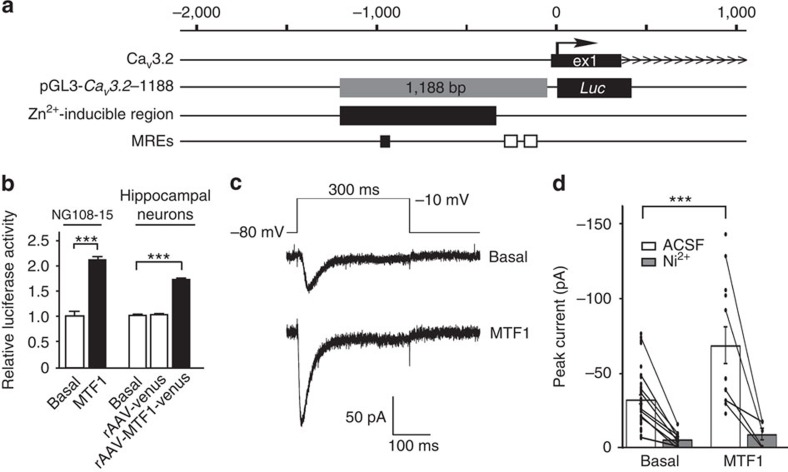
MTF1 mediates the activation of *Ca*_*V*_*3.2* promoter by Zn^2+^ and increases functional expression of Ca_V_3.2. (**a**) Schematic overview of the rat *Ca*_*V*_*3.2* promoter region with the *Ca*_*V*_*3.2-*1188 promoter–luciferase reporter construct^20^. The identified Zn^2+^-inducible region is indicated (black box) together with the three MREs. One MRE is located within the Zn^2+^-inducible region (black bar), whereas two MREs are located outside the Zn^2+^-inducible region (white bars). (**b**) Left panel: luciferase activity of the *Ca*_*V*_*3.2* promoter construct after transfection with MTF1 in NG108-15 cells. Right panel: luciferase activity of the *Ca*_*V*_*3.2* promoter in rat hippocampal neurons transduced with rAAV particles harbouring the rat *Ca*_*V*_*3.2* promoter luciferase reporter (rAAV-*Ca*_*V*_*3.2*) (ref. 39), a pRL-TK control promoter construct, an expression construct for MTF1 (rAAV-CMV-MTF1-IRES-Venus) or a control expression construct (rAAV-CMV-Venus) at DIV1 and measured at DIV15 (*t-*test: ***P≤0.001; *n*=3). (**c**) Ca^2+^ currents in NG108-15 cells were elicited with a voltage step from −80 to −10 mV (upper part). Representative current traces show an increased amplitude in MTF1-transfected cells (lower trace) compared with controls (upper trace). (**d**) Average of the transient Ca^2+^ currents for control cells (*n*=37) and cells transfected with MTF1 (*n*=12) display the functional upregulation of T-type Ca^2+^ currents (*t*-test: ***P≤0.001). Average of the transient currents after application of 100 μM Ni^2+^ showed the large amplitude reduction in all recorded cells (*n*=9 for basal conditions and *n*=5 following Zn^2+^ application), indicating involvement of Ca_V_3.2 channels.

Subsequent bioinfomatic analyses revealed a surprising accumulation of potential binding sites (metal-responsive elements (MREs)) for MTF1 (Table 1)^22^. One of the bioinformatically detected MREs resided within the above identified Zn^2+^-inducible minimal *Ca*_*V*_*3.2* promoter region (Fig. 4a), supporting a role for MTF1 in the Zn^2+^-dependent Ca_V_3.2 mRNA expression. To test if MTF1 indeed is able to stimulate the *Ca*_*V*_*3.2* promoter, we analysed *Ca*_*V*_*3.2* promoter activity after MTF1 overexpression in NG108-15 cells and primary hippocampal neurons. Indeed, MTF1 overexpression increased *Ca*_*V*_*3.2* promoter activity in both cell types to similar levels as observed after stimulation with K^+^+Zn^2+^ (Fig. 4b). Whole-cell patch-clamp recordings revealed that overexpression of MTF1 in NG108-15 cells led to a substantial increase of *I*_CaT_ (Fig. 4c). These augmented currents were almost completely blocked by 100 μM Ni^2+^ (Fig. 4d), indicating that they are generated by Ca_v_3.2 subunits.

### MTF1 binds a Zn^2+^-sensitive MRE in the *Ca*_*V*_ *3.2* promoter

**Figure 5:**
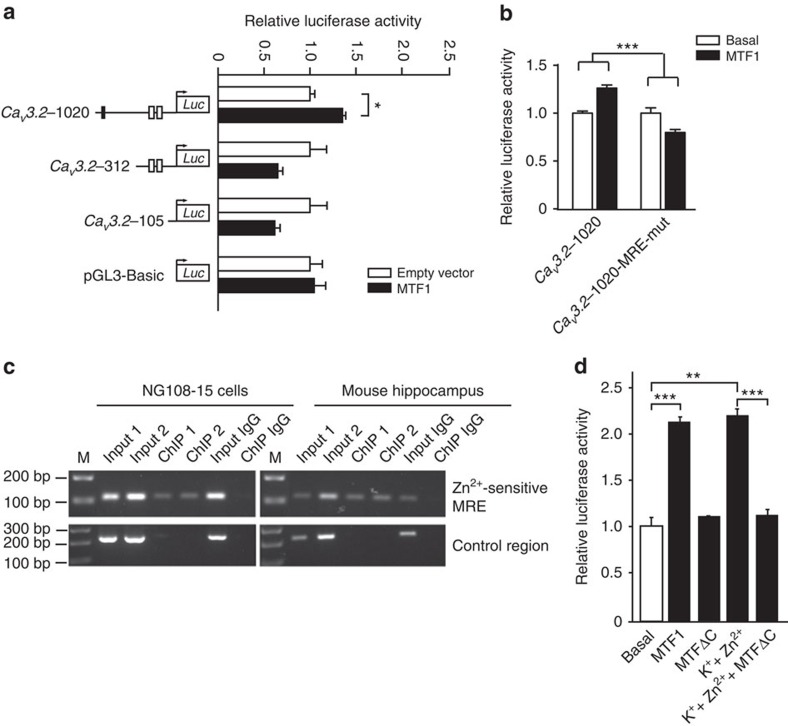
MTF1 binds the *Ca*_*V*_*3.2* promoter. (**a**) Luciferase activity of the *Ca*_*V*_*3.2* promoter deletion fragments after overexpression with MTF1. Note the almost similar activation pattern of the *Ca*_*V*_*3.2* promoter deletion fragments as seen after stimulation with K^+^+Zn^2+^ (50 mM/200 μM; Fig. 3c). No activation was observed for the pGL3-basic control plasmid (one-way analysis of variance (ANOVA): *P*=0.012; *F*_(7,16)_=3.856; Tukey's multiple comparisons test, *P≤0.05; *n*=3). (**b**) Luciferase activity of the *Ca*_*V*_*3.2*-1020 fragment and the *Ca*_*V*_*3.2*-1020 fragment with the mutated Zn^2+^-sensitive MRE-binding site (*Ca*_*V*_*3.2*-1020-MRE-mut) after overexpression with MTF1. Mutation of the Zn^2+^-sensitive MRE-binding site resulted in a reduced *Ca*_*V*_*3.2* promoter activity (two-way ANOVA: *P*=0.0002; *F*_(1,8)_=38.9. (**c**) ChIP analysis of MTF1 binding to the Zn^2+^-sensitive MRE within the *Ca*_*V*_*3.2* promoter. PCR amplicons were generated of anti-MTF1 ChIP immunoprecipitates from NG108-15 cells and mouse hippocampi, using primer pairs spanning the Zn^2+^-sensitive MRE and a control region in the *Ca*_*V*_*3.2* promoter lacking a MRE. A rabbit-IgG immunoprecipitate was used as negative control. (**d**) Luciferase activity of unstimulated and K^+^+Zn^2+^-challenged (50 mM/200 μM) NG108-15 cells transfected with the full-length *Ca*_*V*_*3.2* promoter–luciferase reporter construct and MTF1 or MTF1ΔC (one-way ANOVA: *P*<0.001; *F*_(4,10)_=117.9; Tukey's multiple comparisons test, **P≤0.01, ***P≤0.001; *n*≥3).

We then probed whether the Zn^2+^-inducible genomic fragment of the *Ca*_*v*_*3.2* promoter (Fig. 4a) corresponds to the genomic region of the *Ca*_*v*_*3.2* promoter with maximal MTF1 responsiveness. We therefore analysed the activity of three *Ca*_*V*_*3.2* promoter deletion fragments in the presence of MTF1. Only the *Ca*_*V*_*3.2* promoter fragment harbouring the MRE located in the Zn^2+^-inducible region was activated by MTF1 overexpression (Fig. 5a). Subsequent mutation of this MRE significantly reduced the *Ca*_*V*_*3.2* promoter activity after MTF1 overexpression (Fig. 5b), indicating that this MRE is indeed responsible for the MTF1-induced *Ca*_*V*_*3.2* upregulation. In addition, chromatin immunoprecipitation (ChIP) analysis of NG108-15 cells, as well as of mice hippocampi, revealed binding of MTF1 to the Zn^2+^-sensitive MRE in the *Ca*_*V*_*3.2* promoter region (Fig. 5c).

To prove unequivocally that the Zn^2+^-induced *Ca*_*V*_*3.2* promoter activation is mediated by MTF1, a dominant-negative form of MTF1 (MTF1ΔC)^23^ was co-transfected with the *Ca*_*V*_*3.2*-1188 reporter plasmid. MTF1ΔC is still able to bind MREs but does not possess the ability to activate transcription, thereby blocking MREs from wild-type MTF1. Treatment with K^+^+Zn^2+^ solution of cells overexpressing MTF1ΔC resulted in a complete repression of Zn^2+^-induced *Ca*_*V*_*3.2* promoter activation (Fig. 5d). Therefore, MTF1 appears necessary and sufficient to mediate the stimulatory effects of Zn^2+^ on the *Ca*_*V*_*3.2* promoter.

### MTF1 upregulates hippocampal Ca_V_3.2 mRNA *in vivo*

**Figure 6:**
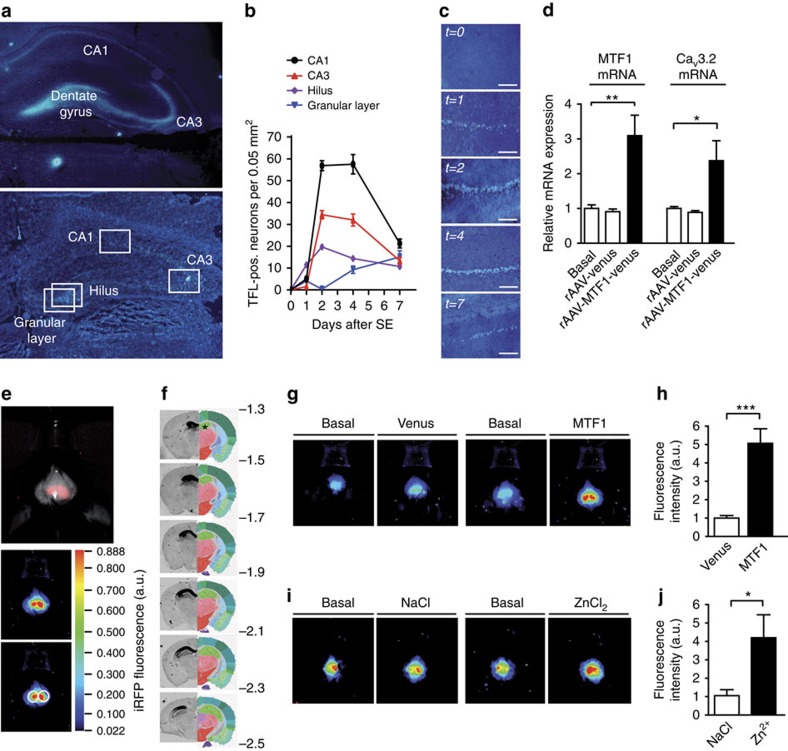
The Zn^2+^–MTF1–Ca_V_3.2 cascade contributes to Ca_V_3.2 upregulation. (**a**) TFL-Zn^2+^ staining of the hippocampal region in the pilocarpine-SE model in rats under basal conditions (upper panel) and 2 days after SE (lower panel). White boxes represent the grids for differential counting in four distinct areas (CA1, CA3, hilus and the granular layer of the dentate gyrus (DG)). (**b**) Quantification of TFL-positive neurons in hippocampal CA1, CA3 and DG at various time points after SE (*n*=3 rats per time point; 39 slices). (**c**) Representative examples of TFL-Zn^2+^ staining in the CA1 area under basal conditions and 1, 2, 4 and 7 days after SE. Scale bar, 50 μm. (**d**) Quantitative RT–PCR on total hippocampi isolated from control, rAAV-Venus and rAAV-MTF1-Venus-injected mice. Expression levels were measured 14 days after injection, with synaptophysin as reference gene (one-way analysis of variance: *P*<0.001; *F*_(5,18)_=12.32; Tukey's multiple comparisons test, *P≤0.05, **P≤0.01; *n*=3). (**e**) Near-infrared *in vivo* imaging. Upper panel: representative example of *in vivo* iRFP signal of a recorded mouse with exposed skull. Middle panel: pseudo colour visualization of iRFP signals. Lower panel: regions of interest (ROIs) were defined above the hippocampal region and the surface radiance was defined in arbitrary units (a.u.). The colour bar indicates the total fluorescence efficiency. (**f**) iRFP fluorescence of coronal brain slices isolated from a rAAV-*Ca*_*V*_*3.2*-iRFP-injected animal (left side: iRFP fluorescence intensity; right side: reference images from the Allen Mouse Brain Atlas (©2015 Allen Institute for Brain Science: http://mouse.brain-map.org), with the hippocampal region annotated with an asterisk (upper panel). Positions (in mm) are given relative to Bregma. (**g**) Representative example of a recorded rAAV-*Ca*_*V*_*3.2*-iRFP mouse under basal conditions and 3 weeks after injection with rAAV-Syn-Venus (Venus) or rAAV-Syn-MTF1-IRES-Venus (MTF1). (**h**) Quantification of iRFP signals of rAAV-Syn-Venus (*n*=10)- and rAAV-Syn-MTF1-IRES-Venus (*n*=10)-injected animals (*t-*test: ***P≤0.001). (**i**) Representative example of a recorded rAAV-*Ca*_*V*_*3.2*-iRFP mouse under basal conditions and 3 days after injection with 1 μl 0.9% NaCl or 1 μl 100 μM ZnCl_2_. (**j**) Quantification of iRFP signals of NaCl (*n*=8)- and ZnCl_2_ (*n*=8)-injected animals (*t-*test: *P≤0.05).

It was previously shown that pilocarpine-SE induces an increase in [Zn^2+^]_i_ in the somata of CA1 pyramidal cells^14^, likely due to the release of intracellularly bound Zn^2+^ (ref. 24). We investigated in rats the time course of this increase using TFL staining of hippocampal slices resected from control and SE-experienced animals (Fig. 6a; CA1, CA3, hilus and granular layer of the dentate gyrus). The strongest increase in TFL staining was observed in the CA1 region (Fig. 6b). TFL-positive pyramidal cells first appeared at 1 day after SE, their incidence peaking 2–4 days after SE and declining thereafter (Fig. 6b,c). These data suggest an association between the SE-induced rise in [Zn^2+^]_i_ and the SE-induced increases in Ca_V_3.2 mRNA and protein, as well as in *I*_CaT_ (ref. 13). To test whether MTF1 mediates between the rise in [Zn^2+^]_i_ and associated increases in Ca_V_3.2 expression and function, we first transduced mice brains with MTF1. To that end, we injected either rAAV-CMV-MTF1-IRES-Venus (MTF1 group) or rAAV-CMV-Venus particles (control group) into area CA1 of mice hippocampi. The mRNA isolated from hippocampi of both groups revealed significantly increased MTF1 mRNA expression in the former group, indicating efficient viral transduction. Correspondingly, we observed Ca_V_3.2 mRNA expression to be significantly increased in the MTF1 group versus the control group (Fig. 6d). In addition, *in vivo* imaging using infrared fluorescent proteins (iRFP) under control of the *Ca*_*V*_*3.2* promoter (Fig. 6e,f) also showed the activation of the *Ca*_*V*_*3.2* promoter after transduction with MTF1 and a remarkably similar activation after intrahippocampal injection of Zn^2+^ (Fig. 6g–j). These data thus show that MTF1 and Zn^2+^ activate the *Ca*_*V*_*3.2* promoter also *in vivo*.

**Figure 7:**
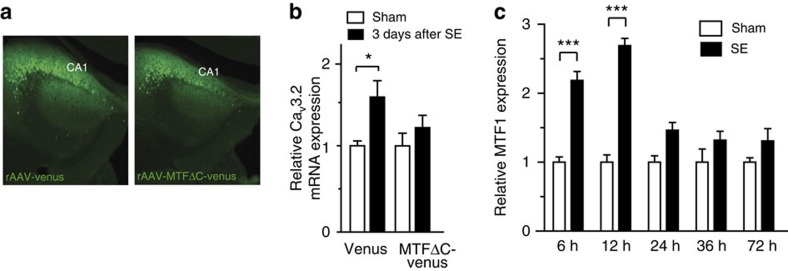
The Zn^2+^–MTF1–Ca_V_3.2 cascade in the pilocarpine-SE model. (**a**) Representative image of a rAAV-Venus- and rAAV-MTF1ΔC-Venus-injected animal. (**b**) mRNA expression of Ca_V_3.2 in mice injected with rAAV-Venus (left) and rAAV-MTF1ΔC-Venus (right) 3 days after pilocarpine-induced SE (quantification based on synaptophysin; one-way analysis of variance (ANOVA): *P*=0.0335; *F*_(3,26)_=3.374; Tukey's multiple comparisons test, *P≤0.05; *n*=4). (**c**) MTF1 mRNA expression of hippocampal CA1 6, 12, 24, 36 and 72 h after pilocarpine-induced SE in mice (quantification based on synaptophysin; one-way ANOVA: *P*<0.001; *F*_(9,39)_=21.71; Tukey's multiple comparisons test, ***P≤0.001; *n*≥4).

We next similarly injected mice with MTF1ΔC. We injected either rAAV-CMV-MTF1ΔC-IRES-Venus (MTF1ΔC group) or rAAV-CMV-Venus particles (control group) into area CA1 of mice hippocampi (Fig. 7a). Two weeks after injection, mice were either subjected to pilocarpine-SE or sham treated. The mice were killed 3 days thereafter, that is, at the time point of maximal Ca_V_3.2 mRNA increase after SE^13^. Interestingly, overexpression of MTF1ΔC significantly reduced the SE-induced increase in Ca_V_3.2 mRNA peak during early epileptogenesis (Fig. 7b), further indicating that MTF1 mediates between the SE-induced rise in [Zn^2+^]_i_ and the transcriptional Ca_V_3.2 upregulation.

Given its key role in SE-induced Ca_V_3.2 transcription, we also studied whether pilocarpine-SE affects MTF1 expression levels. We found that the levels of MTF1 mRNA significantly increased 6 and 12 h after SE, and returned to basal values thereafter (Fig. 7c).

### Interfering with MTF1 attenuates seizure development

**Figure 8:**
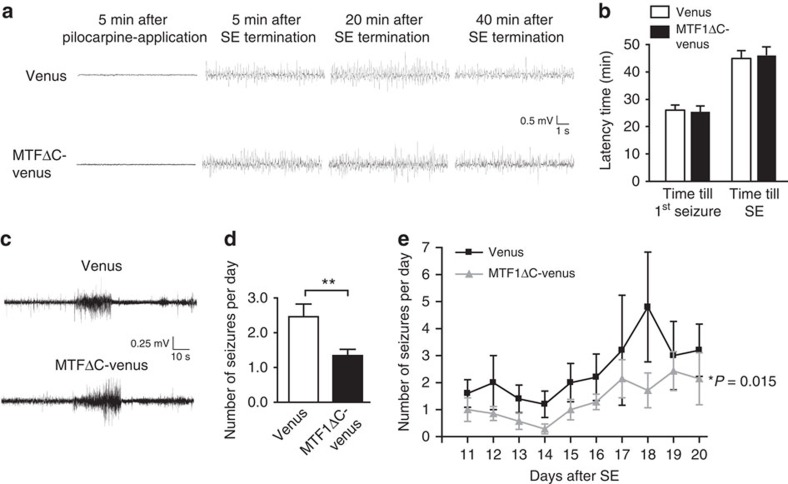
Emergence of spontaneous seizures is markedly attenuated in animals transduced with MTF1ΔC. (**a**) Representative EEG traces of a rAAV-Venus- and rAAV-MTF1ΔC-Venus-injected animal 5 min after pilocarpine application and 5, 20 and 40 min after SE onset. (**b**) No differences for the latency to first seizure (left) and latency to SE (right) in animals injected with rAAV-MTF1ΔC-Venus (*n*=7) compared with animals injected with rAAV-Venus (*n*=5). (**c**) Representative EEG recordings from SE-experienced rAAV-Venus- and rAAV-MTF1ΔC-Venus-injected animals. (**d**) Average number of seizures in the chronic epileptic stage in rAAV-Venus- (*n*=5) and rAAV-MTF1ΔC-Venus-injected animals (*n*=7; *t-*test: **P≤0.01). (**e**) Spontaneous seizure activity after SE in rAAV-Venus versus rAAV-MTF1ΔC-Venus animals. The frequency of spontaneous seizures is significantly decreased in rAAV-MTF1ΔC-Venus (*n*=5) versus rAAV-Venus animals (*n*=7; *t-*test: *P≤0.05).

We have previously shown that mice lacking Ca_V_3.2 manifested a much milder form of chronic TLE^13^. We therefore expected that interfering with the Zn^2+^–MTF1–Ca_V_3.2 cascade would also exert an antiepileptogenic action. We tested this prediction in mice transduced with MTF1ΔC as described above. With respect to the acute SE induced by pilocarpine, we found no differences in electroencephalography (EEG) recordings between MTF1ΔC mice and control animals before, during and after the SEs (Fig. 8a). Likewise, the two groups of mice were similar with respect to the latencies to the first acute seizure and to the onset of SE (Fig. 8b). These results show that the two groups of mice likely experience SEs of identical intensities. Further EEG monitoring indicated that spontaneous seizures emerged in both groups of mice (representative examples of EEGs shown in Fig. 8c). However, seizure frequency was substantially lower in MTF1ΔC mice compared with control animals (Fig. 8d,e). Intriguingly, the extent of neurodegeneration found in MTF1ΔC mice was similar to that found in control animals, despite the lesser seizure frequency in the former group (Supplementary Fig. 3).

### Increased MTF1 and Ca_V_3.2 expression correlate in HS

**Figure 9:**
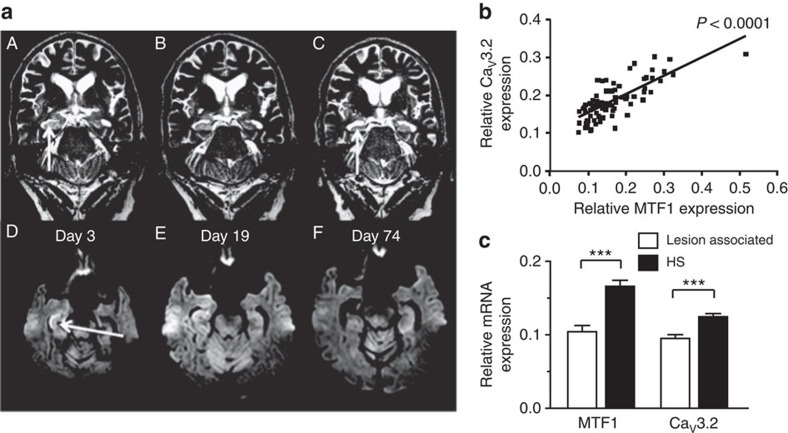
MTF1 and Ca_V_3.2 expression levels co-segregate and are increased in hippocampal tissue of patients with HS. (**a**) Epileptogenesis in a human individual without any previous neurological symptoms. The patient initially manifested clinically with SE (Supplementary Note 2). Coronal T2-weighted fast spin echo (A–C) and axial diffusion-weighted spin echo planar imaging (EPI) show the rapid development of a right-sided HS in the clinical course. Initially, there is hippocampal swelling (A: arrow) associated with cytotoxic oedema of the CA1 sector (D: arrow). Two weeks later, swelling and cytotoxic oedema are somewhat regredient but still present (B,E). Only 8 weeks later, cytotoxic oedema has disappeared (F) and hippocampal atrophy, that is, HS has manifested (C: arrow). MNI (Montreal Neurological Institute) coordinates as derived from the ‘standard brain template' correspond to 30, −14 and 20. (**b**) Regression analyses of Ca_V_3.2 mRNA versus MTF1 mRNA expression in patients with HS. A strong positive correlation between the two variables is present even in the heterogeneous group of human HS hippocampi. (**c**) Quantitative determination of MTF1 and Ca_V_3.2 mRNA. MTF1 and Ca_V_3.2 are significantly abundant expressed in hippocampal tissue of TLE patients with HS versus hippocampi from patients with lesion-associated TLE, that is, in which seizures are explained by lesions such as low-grade neoplasms and/or focal dysplasia in the immediate vicinity or even including the hippocampal formation (HS: *n*=79; lesion associated: *n*=35; *t-*test: ***P≤0.001, with synaptophysin as reference gene).

To assess the potential role of the Zn^2+^–MTF1–Ca_V_3.2 cascade in human TLE, we analysed hippocampal MTF1 and Ca_V_3.2 expression levels in pharmacoresistant TLE patients with hippocampal sclerosis (HS) versus patients with ‘lesion-associated' TLE (Supplementary Note 1). We found a strong positive correlation between MTF1 and Ca_V_3.2 expression levels in both groups of patients (Fig. 9b, Supplementary Fig. 4). These data indicate that the correlation of expression between MTF1 and Ca_V_3.2 is a rather stable phenomenon, especially when considering patients heterogeneity with respect to endophenotypes (for example, hippocampal damage, time point after seizure onset, etc.) and genetic background. Intriguingly, both MTF1 and Ca_V_3.2 mRNA expression levels were substantially higher in TLE patients with HS compared with those with ‘lesion-associated' TLE (Fig. 9c). This difference may reflect differences in seizure frequencies or intensities between the two groups or in other factors regulating [Zn^2+^]_i_.

## Discussion

Here we describe a novel mechanism of neuronal plasticity, which we refer to as the Zn^2+^–MTF1–Ca_V_3.2 cascade, wherein a rise in [Zn^2+^]_i_ activates MTF1, which then binds to MREs in the *Ca*_*V*_*3.2* gene promoter and increases transcription of this gene. The increase in Ca_V_3.2 mRNA leads to enhanced expression of Ca_V_3.2 channels and larger *I*_CaT._ In CA1 pyramidal cells, the *I*_CaT_ increase causes regular firing cells to convert to burst firing, thereby enhancing the excitability of the hippocampal network^10^,^11^. Our cumulative data suggest that this cascade may play a pivotal role in epileptogenesis triggered by pilocarpine-SE. First, pilocarpine-SE leads to a rise in [Zn^2+^]_i_ in CA1 pyramidal cells, appearing within a day and persisting for at least 1 week. Second, exposure of cells to elevated [Zn^2+^]_i_ induces transcriptional upregulation of Ca_V_3.2 mediated by MTF1. Third, pilocarpine-SE causes a selective increase in *I*_CaT_ in CA1 pyramidal cells^12^ that is underlain by transcriptional upregulation of Ca_V_3.2 (ref. 13). This increase is temporally correlated with the rise in [Zn^2+^]_i_ and is reduced by transfection of these neurons with MTF1ΔC (shown to interfere with Zn^2+^-induced Ca_V_3.2 mRNA upregulation), suggesting a causal relationship between the two SE-induced effects. Finally, deletion of Ca_V_3.2 markedly attenuates the emergence of recurrent seizures following pilocarpine-SE^13^, and this antiepileptogenic effect is mimicked by transfecting the hippocampi with MTF1ΔC. Thus, the development of chronic TLE in the pilocarpine-SE model may be attenuated by targeting different components of the Zn^2+^–MTF1–*Ca*_*V*_*3.2* cascade that lead to Ca_V_3.2 upregulation.

We found that [Zn^2+^]_i_ increases, acting via MTF1, upregulate *Ca*_*V*_*3.2* promoter activity but do not affect transcription of other T-type Ca^2+^ channel subunits. This intriguing selectivity is due to the fact that only Ca_V_3.2, but not Ca_V_3.1 and Ca_V_3.3, contain MREs in the 1.5-kb genomic region upstream of the start ATG. Indeed, gene promoters that harbour MREs are sparsely distributed throughout mammalian genomes^25^. The only ones known to be regulated by MTF1 in a [Zn^2+^]_i_-dependent manner are the genes encoding for metallothioneins. These are small, cysteine-rich proteins with a high affinity for Zn^2+^ and other heavy metals^26^. By increasing the expression of metallothioneins, MTF1 acts in a negative feedback manner to facilitate removal of excess free Zn^2+^ (ref. 27).

In the pilocarpine-SE model, deleting *Ca*_*V*_*3.2* not only reduced the frequency of recurrent seizures in the chronic stage but also strongly protected the hippocampus from SE-induced neurodegeneration^13^. Here we show that transducing hippocampi with MTF1ΔC also reduced chronic seizures frequency, but did not prevent neurodegeneration. This discrepancy may be due to the fact that MTF1ΔC overexpression also interferes with MTF1-mediated upregulation of metallothioneins, thereby allowing [Zn^2+^]_i_ to increase to toxic levels. To overcome this side effect, and to unequivocally prove that the reduction of seizures after treatment with MTF1ΔC is due to a direct effect on the *Ca*_*V*_*3.2* promoter, the Zn^2+^-sensitive MRE in the *Ca*_*V*_*3.2* promoter could be genetically modified *in vivo* using the CRISPR/Cas complex^28^. Subsequent analysis of these mice in the pilocarpine-SE model will then reveal whether they experience less seizure activity as well as a reduced neurodegeneration.

Our findings suggest that pilocarpine-SE evokes the Zn^2+^–MTF1–Ca_V_3.2 cascade by inducing a rise in [Zn^2+^]_i_. However, the mechanism coupling SE to [Zn^2+^]_i_ increase is yet unknown. During the intense neuronal activity underlying SE, labile Zn^2+^ is released from glutamatergic terminals and may enter postsynaptic neurons via multiple routes^14^. Alternatively, Zn^2+^ tightly bound to metallothioneins may be released by action of nitric oxide^29^,^30^, whose production is markedly increased during SE^31^. In either case, another potential strategy to impede the Zn^2+^–MTF1–Ca_V_3.2 cascade would be the early application of cell permeable Zn^2+^ chelators. However, given that Zn^2+^ is mandatory for many critical cell processes^32^, its chelation might lead to deleterious effects^33^. An alternative strategy to impede the Zn^2+^–MTF1–Ca_V_3.2 cascade would be to interfere with nitric oxide accumulation by application of nitric oxide synthase inhibitors or scavengers. Pharmacological targeting of Ca_V_3.2 blockers early after pilocarpine-SE may also prove to be antiepileptogenic, and selective Ca_V_3.2 blockers are becoming available^34^. Alternatively, MTF1 may be targeted pharmacologically. The fact that activation and promoter binding of MTF1 require phosphorylation^23^,^35^ may enhance the development of inhibitory drugs, for example, by small molecule library screening^36^. As perspective, we suggest that pharmacological interventions targeting the Zn^2+^–MTF1–Ca_V_3.2 cascade may prove as intriguing future option for treating pharmacoresistant TLE.

## Methods

### Bioinformatic analysis and plasmids

MREs were identified using the software tool PoSSuMsearch^37^ with position-specific scoring matrices from the TRANSFAC database^38^.

The mammalian expression vectors pCDNA3-HA-mMTF1 and pCDNA3-MTF1-EcoRl (dominant negative; MTF1ΔC) were kindly provided by Carl Seguin (Québec) and Guy J. Rosnan (Fred Hutchinson Cancer Center, Seattle).

The *Ca*_*V*_*3.2*-1020-MRE-mut reporter plasmid was made by mutating the Zn^2+^-sensitive MRE in the *Ca*_*V*_*3.2*-1020 luciferase promoter fragment^20^. For this, the first three nucleotides of the MRE consensus sequence (TGC; Table 1) were mutated into GAG, resulting in a destroyed MRE. Mutagenesis was performed using the QuikChange II XL Site-Directed Mutagenesis Kit (Agilent Technologies, Waldbronn, Germany) with the following primers: 5′-CACTGCGGGGGCCTCGAGCCGGCGGGG-3′ and 5′-CCCCGCCGGCTCGAGGCCCCCGCAGTG-3′, and with conditions as follows: 2 min at 95 °C, then 18 cycles of 50 s at 95 °C, 50 s at 55 °C and 10 min at 68 °C.

For construction of the adeno-associated viral (AAV) plasmids, pAAV-CMV-MCS harbouring the AAV2 inverted terminal repeats (Stratagene, La Jolla, USA) was modified. For pAAV-CMV-MTF1-IRES-Venus, first an IRES-Venus sequence was cloned in the SalI and BglII sites of pAAV-CMV-MCS. The MTF1 sequence was amplified from pCDNA3-HA-mMTF1 using primers with Bsu15I and BglII overhang and cloned in the Bsu15I and BamHI digested pAAV-CMV-IRES-Venus vector, resulting in pAAV-CMV-MTF1-IRES-Venus. For pAAV-CMV-Venus, a sequence encoding the green fluorescent protein Venus was inserted in the HindIII and BglII sites of pAAV-CMV-MCS. The pAAV-hSyn-Venus construct was made from pAAV-CMV-Venus by exchange of the promoters via the MluI and Bsu15I sites. The pAAV-hSyn-MTF1C-IRES-Venus construct was made from pAAV-CMV-IRES-Venus by exchange of the CMV with hSynapsin promoter (MluI and EcoRI) and introducing MTF1ΔC (EcoRI and BglII/BamHI).

The pAAV-*Ca*_*V*_*3.2*-luciferase construct has been described previously^39^. For the AAV-RL-TK control plasmid, the RL-TK cassette (Promega, Mannheim, Germany) was amplified with NotI overhang and cloned in NotI-digested pAAV-MCS. The pAAV-*Ca*_*V*_*3.2*-iRFP^713^ was made by exchanging the luciferase from pAAV-*Ca*_*V*_*3.2*-venus with iRFP^713^ (Addgene clone #31857) using HindIII and BglII restriction sites. All AAV-cloning procedures were performed in Stbl2 bacteria (Life Technologies, Germany) to minimize recombination events. Plasmid sequences were verified by sequencing analysis. Integrity of the inverted terminal repeats was confirmed by SmaI restriction analysis.

### Cell cultures

Several types of cultured cells were used in this study. NG108-15 cells (American Type Culture Collection HB-12317) were maintained at 37 °C and 5% CO_2_ in DMEM supplemented with 10% (v/v) heat-inactivated FCS (Invitrogen) 100 units per ml penicillin/streptomycin, 2 mM glutamine and 1 × HAT (sodium hypoxanthine, aminopterin and thymidine; Invitrogen). If not stated otherwise, NG108-15 cells were seeded in 24-well plates with 60,000 cells per well. HEK293 cells stably transfected with human Ca_V_3.2 (kindly provided by Ed Perez-Reyes, University of Virginia, Charlottesville, VA, USA) and HEK293-AAV cells (#240073, Stratagene, La Jolla, CA) were kept in high-glucose DMEM supplemented with 10% FCS (Invitrogen), 100 units per ml penicillin/streptomycin and 2 mM glutamine, and incubated at 37 °C and 5% CO_2_. Primary rat hippocampal neurons were prepared and kept in culture as described previously^40^.

### Zn^2+^ loading of NG108-15 cells

Twenty-four hours after seeding, NG108-15 cells were loaded for 30 min with calcein red-orange AM (2.5 μg per well; Invitrogen Molecular Probes). Next, the cells were incubated for 4 h with one of the following solutions: (i) basal solution containing (in mM): NaCl, 140; KCl, 3; CaCl_2_, 2; MgCl_2_, 1; D-glucose, 25; HEPES/NaOH, 10 (pH 7.4); (ii) K^+^ solution, same as the basal solution but KCl concentration raised to 50 mM; (iii) Zn^2+^ solution, same as the basal solution but with added Zn^2+^ (200 μM); and (iv) K^+^+Zn^2+^ solution, same as the K^+^ solution but containing also 200 μM Zn^2+^. Fifteen minutes after returning the cells to the DMEM incubation medium they were exposed to the fluorescent Zn^2+^ indicator *N*-(6-methoxy-8-quinolyl)-*p*-toluenesulfonamide (TSQ; 0.001% final, added from 0.5% weight per volume stock in dimethylsulfoxide). Ten minutes later, cells were examined and photographed under a fluorescence microscope (Axio Observer.A1, Zeiss). Photographs ( × 20) were taken under identical conditions. Background-corrected calcein red-orange and TSQ fluorescence was quantified for single cells (regions of interest were set using a differential interference contrast image) using ImageJ software (NIH) and averaged for every field of view.

### mRNA isolation and real-time RT–PCR quantification

mRNA from NG108-15 cell preparations and hippocampi was isolated using the Dynabeads mRNA Direct Micro Kit (Invitrogen) according to the manufacturer's protocol. cDNA was synthesized by reverse transcription from total RNA using the RevertAid Premium First strand cDNA Synthesis Kit (Fermentas) following the manufacturer's protocol. Ca^2+^ channel subunit transcript quantification was performed by real-time reverse transcription–PCR (RT–PCR). Relative quantification of the starting mRNA copy numbers was carried out according to the ΔΔ*C*_t_ method. The signal threshold was set within the exponential phase of the reaction for determination of the threshold cycle (*C*_t_). PCR samples contained 1 × Maxima Probe/ROX qPCR Master Mix (Fermentas), 5 pM each oligonucleotide primer (Supplementary Table 2) and 1/10 synthesized cDNA in a 6.25-μl volume. Quantitative PCR was performed in an ABI Prism 7900HT apparatus (PE Applied Biosystems, Foster City, CA, USA) with conditions as follows: 2 min at 50 °C, 10 min at 95 °C, then 40 cycles of 15 s at 95 °C and 1 min at 59 °C.

### Western blot analysis

For western blot analysis, cells were washed with ice-cold PBS, detached from the plates and centrifuged for 1 min at 4 °C. Pellets were resuspended in PBS+10 mM EDTA and homogenized by sonification. Proteins were quantified using the nanodrop (ThermoScientific) and 150 μg protein was loaded on 7% Tris-glycine SDS–PAGE gels and transferred to nitrocellulose membranes. Membranes were blocked for 1 h at room temperature in 2% fish gelatin (Sigma) and then incubated for 1 h with antibodies directed against Ca_V_3.2 (1:200; cat #: ACC-025; Alomone Labs) and α-tubulin (1:10,000; ab7291; Abcam). After washing and an hour incubation with the secondary antibodies (IRDye680 goat-anti-rabbit and IRDye800 goat-anti-mouse; both 1:20,000; LI-COR) in PBS+0.1% Tween 20, immunoreactive bands were detected using the Odyssey infrared imaging system (Li-COR Biosciences GmbH, Bad Homburg, Germany) and quantified using the AIDA software (Raytest). The α-tubulin signal was used as internal control. Full blots are shown in Supplementary Fig. 5.

### Electrophysiology

Patch-clamp recordings were obtained from NG108-15 cells. One day after stimulation, cells were washed, trypsinized, seeded on coverslips and measured 4 h after seeding. Patch pipettes (3–4 MΩ) were fabricated from borosilicate glass capillaries and filled with an intracellular solution containing (in mM): CsF, 110; tetraethylammonium chloride, 20; MgCl_2_, 2; HEPES, 10; EGTA, 11; ATP, 5; and GTP, 0.5; pH 7.2 adjusted with CsOH; and osmolality 300 mOsm. Patch-clamp recordings were performed in a bath solution containing (in mM): sodiummethanesulfonate, 125; KCl, 3; MgCl_2_, 1; CaCl_2_, 5; 4-aminopyridine, 4; tetraethylammonium chloride , 20; HEPES, 10; glucose, 10 (pH 7.4, 315 mOsmol). Tight-seal, whole-cell recordings were obtained at room temperature (21–24 °C) according to the standard techniques. Membrane currents were recorded using a patch-clamp amplifier (Axopatch 200B, Molecular Devices, Sunnyvale, USA or EPC9, HEKA Elektronik, Lambrecht, Germany). Series resistance compensation was employed to improve the voltage-clamp control (>80%) so that the maximal residual voltage error did not exceed 1.5 mV. Voltage-clamp recordings were corrected online for a liquid junction potential of 10 mV. Whole-cell Ca^2+^ currents were elicited with depolarizing voltage steps to −10 mV. The magnitude of *I*_CaT_ was quantified by analysing the transient component of the resulting current traces.

The voltage dependence of activation and inactivation was characterized using standard voltage step protocols (Fig. 2a). The voltage-dependent activation of the Ca^2+^ conductance was fit by the product of a Boltzmann function, reflecting voltage-dependent activation equation (1), and the general constant field equation (2):


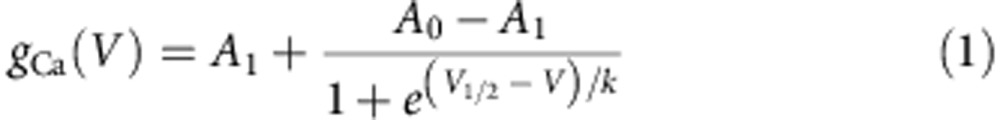



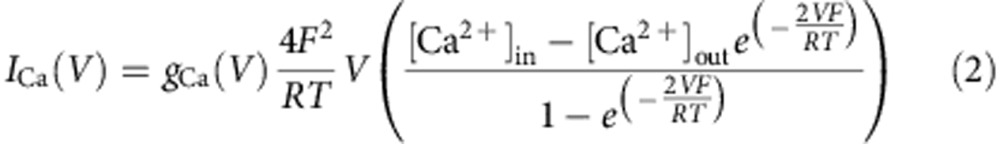


where *I*_Ca_(*V*) denotes the Ca^2+^ current and *g*_Ca_(*V*) the Ca^2+^ conductance amplitude, respectively, at the membrane potential *V* as set by the command voltage *V*. [Ca^*2+*^]_in_ and [Ca^2+^]_out_ correspond to internal and external Ca^2+^ concentration, respectively. The values *V*_1/2_ (membrane potential at half-maximal inactivation or activation), *A*_0_ and *A*_1_ (maximal and minimal conductance, respectively) were determined by the fitting procedure. *F* is Faraday's constant, *R* is the gas constant and *T* is the temperature at which the measurements were conducted (22 °C on average). The conductance *g*_Ca_ for each potential was derived, normalized to *A*_0_ and averaged for all cells of the same group. The voltage dependence of inactivation was derived by converting peak current to *g*_Ca_ and fitting these values with equation (1).

### Luciferase assay

Transfection of the NG108-15 cells was carried out using lipofectamine (Invitrogen) following the manufacturer's protocol. For each well (48-well tissue culture plates; 80% confluency), 0.5 μg *Ca*_*V*_*3.2* luciferase reporter plasmid, 0.0125 μg pRL-TK (Promega) and 0.5 μl lipofectamine were mixed with 25 μl serum-free medium. The mixture was incubated for 20 min at room temperature and then added to the appropriate wells. Cells were grown in serum-free culture medium at 37 °C and 5% CO_2_. After 16 h, the serum-free medium was replaced by serum-containing medium and the cells were used for experiments 36 h after transfection.

*Renilla* luciferase was used to normalize the transfection efficiency data, and a Dual Luciferase Reporter Assay System was used according to the manufacturer's specifications (Promega). *Renilla* and firefly luciferase activities were determined using the Glomax Luminometer (Promega). The results are given as firefly/*Renilla* relative light units if not indicated otherwise.

### Chromatin immunoprecipitation assays

*ChIP on mouse hippocampal tissue.* Mice were decapitated under deep isoflurane anaesthesia (Forene). Hippocampi were removed quickly, snap frozen and stored at −80 °C until further processing. Samples for ChIP experiments were prepared using the SimpleChIP Plus Enzymatic chromatin IP kit (Cell Signaling Technology; #9005) according to the manufacturer's protocol with 5 μg anti-MTF1 (C-19X, SC26844X, Santa Cruz, CA) and 2 μg anti-IgG (Cell Signaling Technology; #2729). The recovered DNA was analysed by PCR with primers spanning the Zn^2+^-sensitive MRE (forward: 5′-CGCGCGAGAAAAGGAGGGGG-3′ and reverse: 3′-GCTCGCAGGGATGCTTGGA-3′) and with primers located in the *Ca*_*V*_*3.2* promoter region, but lacking a MRE (forward: 5′-GAAGGGAGATTCAGCGACAT-3′ and reverse: 5′-CCAATTGTACTGGGGCAGTC-3′). PCR amplification in a 25-μl reaction included 1 μl immunoprecipitated DNA, 10 pmol of each primer and EconoTaq Plus Green 1 × Master Mix (Lucigen Corporation, Middleton, WI). Reactions were amplified for 35 cycles at 94 °C for 30 s, 58 °C for 30 s and 72 °C for 45 s, followed by a final extension step at 72 °C for 10 min. PCR products were analysed on a 2% agarose gel.

*ChIP on NG108-15 cells.* NG108-15 cells (six wells; 100% confluency) were cross-linked with 1% formaldehyde for 10 min at 37 °C. Cells were washed twice in cold PBS containing protease inhibitors (Complete Protease Inhibitor Cocktail Tablets; Roche), lysed in 200 μl SDS lysis buffer (1% SDS; 10 mM EDTA; 50 mM Tris, pH 8.1 with protease inhibitors) and incubated on ice for 10 min. Lysates were sonicated using an Ultrasonic Processor UP50H (Hielscher Ultrasound Technology, Germany), with four sets of 10-s pulses at 50% of maximum power. Samples were centrifuged at 13,000 r.p.m. for 10 min at 4 °C and the supernatant was diluted 10-fold in ChIP dilution buffer (0.01% SDS; 1.1% Triton X-100; 1.2 mM EDTA; 16.7 mM Tris, pH 8.1; and 167 mM NaCl with protease inhibitors). Next, samples were incubated overnight at 4 °C with 5 μg anti-MTF1 (C-19X, SC26844X, Santa Cruz, CA). Rabbit-IgG incubations (Cell Signaling Technology; #2729) were included as control for the immunoprecipitation. Further processing of the ChIP samples was performed using the SimpleChIP Plus Enzymatic chromatin IP kit (Cell Signaling Technology; #9005) as described above.

### Viral vector production

Recombinant AAV1/2 genomes were generated by large-scale triple transfection of HEK293-AAV cells. The rAAV plasmid, helper plasmids encoding *rep* and *cap* genes (pRV1 and pH21), and adenoviral helper pFΔ6 (Stratagene, La Jolla, USA) were transfected using standard CaPO_4_ transfection. Cells were collected ∼60 h following transfection. Cell pellets were lysed in the presence of 0.5% sodium deoxycholate (Sigma) and 50 U ml^−1^ Benzonase endonuclease (Sigma). rAAV viral particles were purified from the cell lysate by HiTrap heparin column purification (GE Healthcare), and then concentrated to a final stock volume of 400 μl using Amicon Ultra Centrifugal Filters (Millipore). Purity of the viruses was validated by coomassie blue staining of SDS–polyacrylamide gels. Functional titres (transducing units) of the fluorescent protein vectors were determined by transduction of cultured primary neurons.

### Animal experiments

*Infusion of AAV vectors.* Mice and rats were housed under a 12 h light/dark cycle with food and water *ad libitum*. All experiments were performed in accordance with the guidelines of the European Union and the University of Bonn Medical Center Animal Care Committee. Adult male mice (∼50 days, >20 g) were obtained from Charles River (C57Bl/6-N) and were anesthetized with 6 mg kg^−1^ xylazine (Rompun; Bayer) plus 90–120 mg kg^−1^ ketamine, intraperitoneal (i.p.) (Ketavet; Pfizer). Intracerebral injection of viral particles in the left and right CA1 hippocampal region was performed stereotactically at the coordinates (in mm) −2 posterior, −2/2 lateral and 1.7 ventral relative to bregma. Holes the size of the injection needle were drilled into the skull, and 1 μl of viral suspension containing ∼10^8^ transducing units was injected using a 10 μl Hamilton syringe at a rate of 100 nl min^−1^ using a microprocessor-controlled mini-pump (World Precision Instruments). After injection, the needle was left in place for 5 min before withdrawal. The needle was then slowly withdrawn and the incision closed.

### Near-infrared *in vivo* imaging

*MTF1 overexpression.* Mice were injected with rAAV-*Ca*_*V*_*3.2*-iRFP particles as described above. Two weeks after injection, mice were anesthetized, the skull was exposed and holes were drilled at the same location for subsequent rAAV-Syn-MTF1-IRES-Venus or rAAV-Syn-Venus injection. Just before injection, basal iRFP values were measured through the skull. Three weeks after injection of viruses harbouring MTF1- or Venus-expressing constructs, iRFP values were again determined.

*Zn^2+^ injection.* Animals were injected with rAAV-*Ca*_*V*_*3.2*-iRFP particles in the hippocampal CA1 region and analysed for their basal iRFP levels 3 weeks after injection. Next, they were injected with 1 μl 100 μM ZnCl_2_ or 1 μl 0.9% NaCl in the same holes as used for the rAAV particles. Animals were imaged again 3 days after injection.

Near-infrared imaging was performed with a Pearl Impulse Small Animal Imaging System (Li-COR Biosciences GmbH). The iRFP signal was determined using a highly sensitive charged-coupled device camera. Excitation and emission wavelengths were fixed at 690 and 710 nm, respectively. Pictures were analysed using the Pearl Impulse Image Studio Software v3.1 (Li-COR Biosciences GmbH). Fluorescent signals were normalized to background levels and quantified by placing two round regions of interest (Fig. 6e) above the hippocampal region^39^. Fluorescent signals are presented as arbitrary units (a.u.).

At the end of the *in vivo* imaging experiments, mice were decapitated under deep isoflurane (Forene) anesthesia. Brains were removed and fixed in formaldehyde overnight. Coronal brain slices (30 μm) were made on a vibratome (Leica), mounted on slides (Histobond, Marienfelde Germany) and imaged on the Odyssey infrared imaging system (Li-COR Biosciences GmbH). Only animals with hippocampal CA1 staining (Fig. 6f) were included in the *in vivo* imaging analysis.

### EEG-video monitoring

The electrographic features of Venus- and MTF1ΔC-injected animals after pilocarpine-SE were analysed with a telemetric EEG/video-monitoring system (Data Science International) as described previously^13^,^41^. Mice were implanted with EEG electrodes directly after the viral injection (described above), when the animals were still narcotized. The transmitter was placed subcutaneously on the right abdominal side and stainless screws were used as electrodes and positioned in the two holes used for the viral injection (2 mm posterior and 2 mm lateral to Bregma). EEG recording with a sampling rate of 1 kHz was started directly after the implantation procedure. Two weeks after the implantation, animals experienced pilocarpine-SE. Monitoring was performed continuously until day 20 after SE. EEG recordings were analysed using NeuroScore v2.1 (DSI) software with the following parameters: a threshold value between 100 and 5,000 μV; a spike duration between 5 and 70 ms; a spike interval between 0.005 and 1 s; a minimum train duration of 5 s; and a minimum number of spikes per event of 10. These parameters were verified by analysing two animals manually for the whole timeframe. All positive events (seizures) of the two animals were identified using these parameters. The output of all animals was checked manually for false-positive events (for example, artefacts) and all false-positive events were deleted from the output files. From concurrent video recordings, all seizures were classified as described previously^41^.

### Pilocarpine-SE in mice and rats

As described previously^41^, adult male C57Bl/6-N mice received a low dose of scopolamine methyl nitrate (1 mg kg^−1^, subcutaneous (s.c.); Sigma) 20 min before the administration of pilocarpine hydrochloride (335 mg kg^−1^, s.c.; Sigma). Forty minutes after SE onset, the mice received 4 mg kg^−1^ s.c. diazepam (Ratiopharm). Sham-control animals were treated identically, but received saline instead of pilocarpine.

Adult male Sabra rats (150–200 g) were kept in the animal facility of Hebrew University and Hadassah School of Medicine. All the experiments were approved by the local institution's ethical committee. Rats were injected with scopolamine methyl nitrate (1 mg kg^−1^, s.c; Sigma). Thirty minutes afterwards, SE was induced with a single dose of pilocarpine (350 mg kg^−1^ (i.p.); Sigma). SE was terminated after 2 h by diazepam (Ratiopharm; 0.1 mg kg^−1^ (i.p.)).

### Zn^2+^ imaging in rat brain slices

Pilocarpine-injected rats were decapitated under isoflurane anesthesia at different time points after the termination of SE. The brains were rapidly extracted and immediately frozen in liquid nitrogen and were stored at −80 °C. Either transversal (2–5.5 mm below the inter-auricular plane, containing the ventral part of the hippocampus) or saggital (1–3.5 mm laterally to the inter-hemispheric plane, containing the dorsal hippocampus) slices, 20-μm wide, were cut in a cryotome and placed on glass slides. For the staining we used 1 of every 10 slices, 200 μm apart from each other, for a total of 15 transverse and 12 sagittal slices for each hemisphere.

For visualization of chelatable Zn^2+^, fresh frozen brain slices were examined under a fluorescent microscope (Olympus BX60, Germany; excitation filter 330–385 nm, emission filter 420 nm), at least 30 min after the preparation. TFL-Zn (Sigma), 250 μM in saline, was applied to the slices, immediately before the microscopic examination, for 30 s and washed with saline. Stained CA1 hippocampal cells were counted by use of a gridded microscope lens (with calculated area of 0.05 mm^2^ in high-power field). The density of Zn^2+^-stained neurons was defined as the number of Zn^2+^-stained neurons counted in a 0.05-mm^2^ area of the slice (grid surface) at high-power field.

### Human TLE patients and mRNA expression analyses

For gene expression analyses, we used human hippocampal biopsy tissue from patients with hippocampal sclerosis (*n*=79) versus patients with lesion-associated (low-grade neoplasms or dysplasia; *n*=35) chronic TLE, who underwent surgical treatment in the Epilepsy Surgery Program at the University of Bonn Medical Center due to pharmacoresistance. In all patients, presurgical evaluation using a combination of non-invasive and invasive procedures revealed that seizures originated in the mesial temporal lobe^42^. All procedures were conducted in accordance with the Declaration of Helsinki and approved by the Ethics Committee of the University of Bonn Medical Center. Informed written consent was obtained from all patients. Clinical characteristics per subgroup are described in Supplementary Table 3. mRNA analyses for Ca_V_3.2 and MTF1 were carried out analogous to a procedure described elsewhere in detail^43^. Briefly, RNA from biopsies representing all hippocampal subfields served to generate 750 ng cRNA used for hybridization on Human HT-12 v3 Expression BeadChips with Illumina Direct Hybridization Assay Kit (Illumina, San Diego, CA) according to standard procedures. We extracted data for Ca_V_3.2 and MTF1 analysed by Illumina's GenomeStudio Gene Expression Module and normalized using Illumina BeadStudio software suite by quantile normalization with background subtraction.

### Statistical analysis

Statistical analyses were performed with GraphPad Prism 6.05 software (GraphPad Software). Sample size (*n*) per experiment was calculated using power analysis, with parameters set within the accuracy of the respective experiment. Student's *t*-tests and repeated measures analysis of variance followed by Bonferroni's multiple comparisons or Tukey's multiple comparisons tests were used to evaluate the statistical significance of the results. Values were considered significantly at *P*<0.05. All results are plotted as mean±s.e.m. All electrophysiological and animal experiments were conducted in a randomized and blinded fashion. All *in vitro* experiments were independently repeated at least two times.

## Additional information

**How to cite this article:** van Loo, K. M. J. *et al.* Zinc regulates a key transcriptional pathway for epileptogenesis via metal-regulatory transcription factor 1. *Nat. Commun.* 6:8688 doi: 10.1038/ncomms9688 (2015).

## Supplementary Material

Supplementary Information Supplementary Figures 1-5, Supplementary Tables 1-3, Supplementary Notes 1-2 and Supplementary References

## Figures and Tables

**Table 1:** MREs located within the *Ca*_*V*_ *3.2* promoter region.

	**Position**	**Orientation**	**Sequence**
1	994 bp	Forward sequence	TGCGCCC
2	275 bp	Forward sequence	TGCGCGC
3	152 bp	Reverse sequence	TGCGCCC
MRE-consensus sequence			TGCRCNC
