# Supplementary material for: Zinc regulates a key transcriptional pathway for epileptogenesis via metal-regulatory transcription factor 1
Source: Nat Commun. 2015 Oct 26;6:8688. doi: 10.1038/ncomms9688 (PMC4846312; doi:10.1038/ncomms9688)
Supplement: Supplementary Information — Supplementary Figures 1-5, Supplementary Tables 1-3, Supplementary Notes 1-2 and Supplementary References [file ncomms9688-s1.pdf]

Supplementary Figures

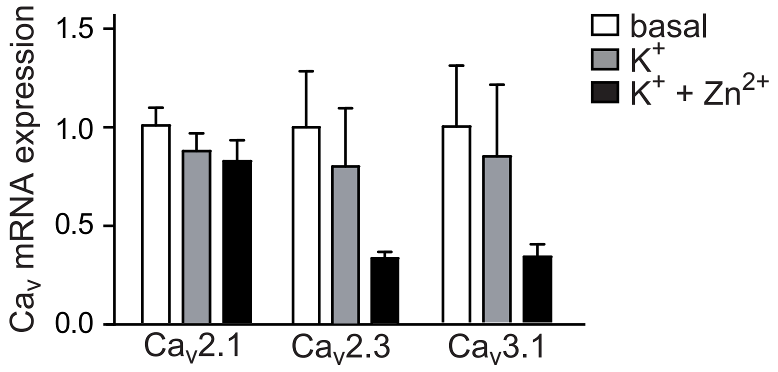

Supplementary Figure 1. Quantitative RT-PCR for the low expressed $Ca_V2.1$, $Ca_V2.3$ and $Ca_V3.1$ mRNAs in NG108-15 cells stimulated with K^+ or K^++Zn^{2+} solutions. mRNA expression was measured 4 hrs after stimulation, with synaptophysin as reference gene (n = 4).

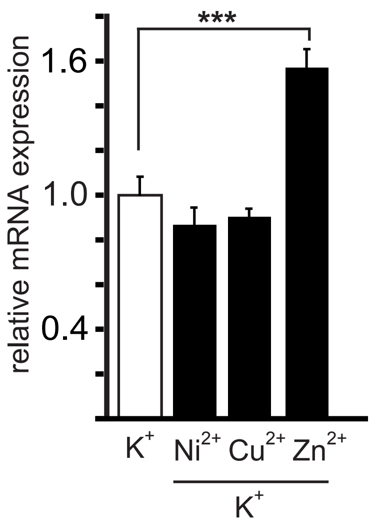

Supplementary Figure 2. Quantitative RT-PCR for $Ca_V3.2$ mRNA levels in NG108-15 cells stimulated with K^+ or K^+ in combination with 500 µM Ni^{2+}, 1 mM Cu^{2+} or 200 µM Zn^{2+}. mRNA expression was measured 4 hrs after stimulation, with synaptophysin as reference gene (One-way ANOVA: ***$P \leq 0.001$; $n = 4$).

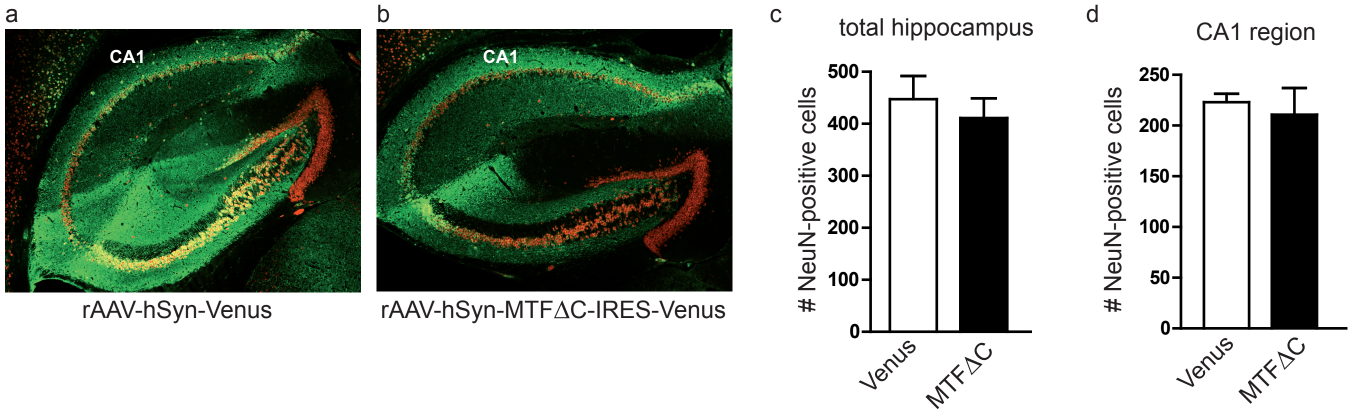

Supplementary Figure 3. The development of hippocampal damage after SE is not altered after transduction with rAAV-hSyn-MTFΔC-IRES-Venus. (**a**) Representative hippocampal section from a rAAV-hSyn-Venus transduced animal and (**b**) rAAV-hSyn-MTFΔC-IRES-Venus transduced animal 30 days after pilocarpine-induced status epilepticus (SE). 4 μm Parafin slices were stained with antibodies directed against the neuron-specific epitope NeuN (1:500, Millipore: MAB377) and Venus (1:1000, Abcam: ab290). (**c**) Number of NeuN-positive cells in the total hippocampus and (**d**) CA1 region of the hippocampus (*n* = 5 animals per group). We did not observe significant differences in cell loss between rAAV-hSyn-Venus and rAAV-hSyn-MTFΔC-IRES-Venus transduced animals in the total hippocampus or the CA1 subregion.

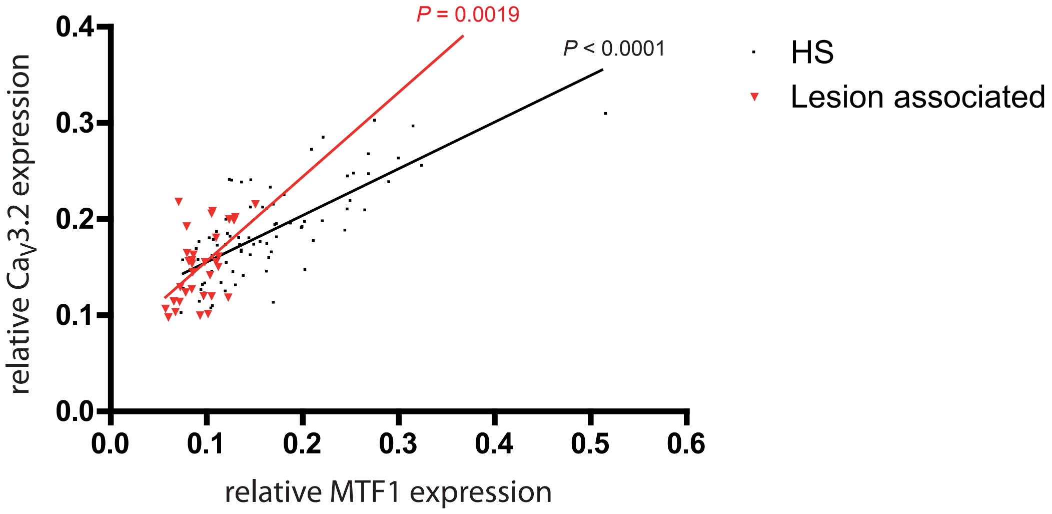

Supplementary Figure 4. Regression analyses of MTF1 mRNA versus $Ca_V3.2$ mRNA expression in lesion associated patients (red triangles) and in patients with hippocampal sclerosis (HS; black dots). A positive correlation between MTF1 and $Ca_V3.2$ expression levels is more evident in the patients with HS.

Supplementary Figure 5. Full blots and gels

Full blot of figure 1f.

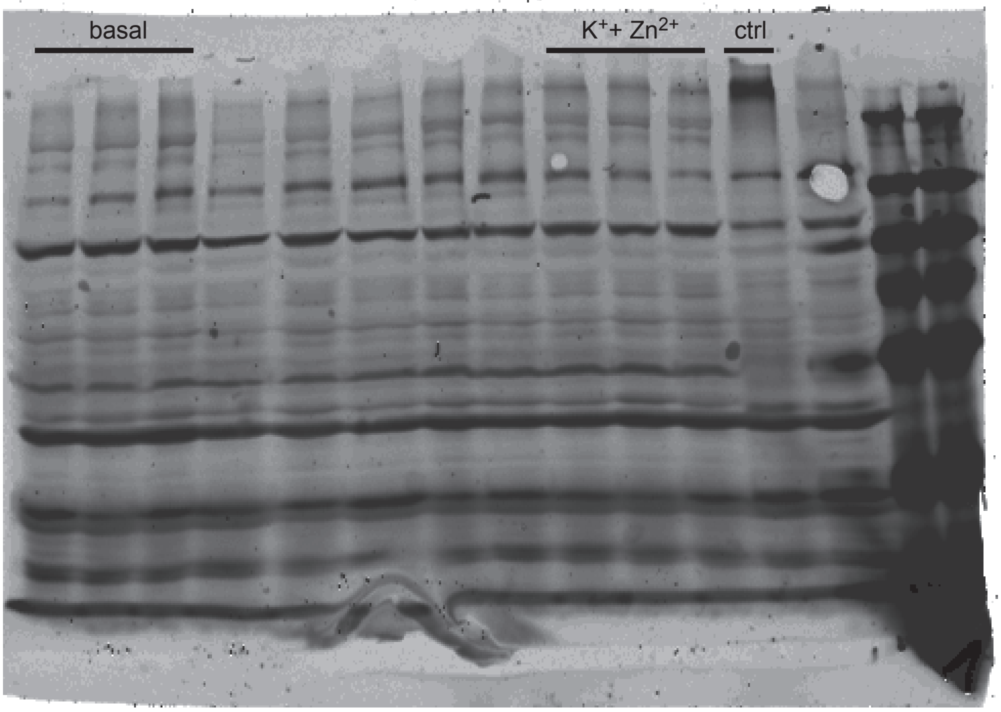

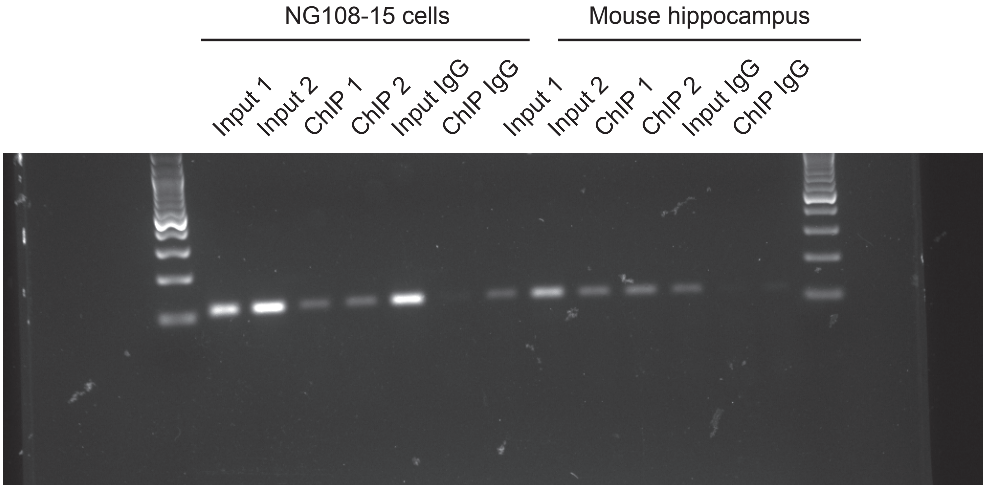

Full gel of figure 5c
(MRE-primer set)

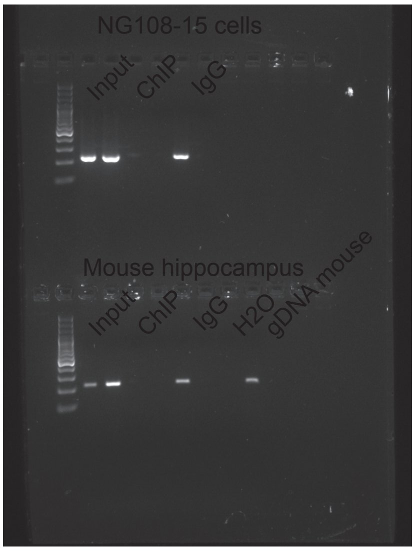

Full gel of figure 5c
(control-primer set)

Supplementary Tables

Supplementary Table 1.

Voltage-dependence $V_{1/2}$ and *k*-values of activation and inactivation after exposing NG108-15 cells to basal and K^++Zn^{2+} solutions

		basal	K^+/Zn^{2+}
activation	$V_{1/2}$ (mV)	-40.7±2.0	-35.3±1.1
	k	6.0±0.2	5.5±0.1
inactivation	$V_{1/2}$ (mV)	-68.3±1.4	-64.6±1.0
	k	5.0±0.4	4.4±0.2

Supplementary Table 2.

Primers sequences for real-time RT-PCR

Gene	Forward Primer	Reverse Primer
Cav1.2	5'-GGAGGACTGGAATTCGGTGAT-3'	5'-ACTAACATCCCTGGAAAAGAGGG-3'
Cav1.3	5'-TTTGACAATGTCCTTTCGGCT-3'	5'-GTTCTCACCGTTTGAATCAATAGCT-3'
Cav2.1	5'-GTCGTGGTGCTAACAGGCATC-3'	5'-ACGAACCGCCCTCAGTGTC-3'
Cav2.2	5'-TCATCGGCCTCGAGTTCTATATG-3'	5'-GGGATCTTTGCCACAGGGA-3'
Cav2.3	5'-GGAGTGGATACCCTTCAATGAGTC-3'	5'-TCTGTTACCACCAGAGATTGTTGTTC-3'
Cav3.1	5'-ACCCTGGCAAGCTTCTCTGA-3'	5'-TTTGCGGAGGATGTACACCAGGT-3'
Cav3.2	5'-TCATCTTCGGCATCGTTGG-3'	5'-CGCAAGAAGGTCAGGTTGTTG-3'
Cav3.3	5'-TCATCCGTATCATGCGTGTTC-3'	5'-GGCCCGCATTCCTGTG-3'
MTF1	5'-AGTGGAGCAGGTGTACTTCGC-3'	5'-TGACAGGCCTCCTCTTGTTTG-3'
Synaptophysin	5'-TTCAGGACTCAACACCTCGGT-3'	5'-CACGAACCATAGGTTGCCAAC-3'

Supplementary Table 3.

Distribution of clinical parameters with the “lesion associated” and hippocampal sclerosis (HS) patient groups

	Lesion associated	(n)	HS	(n)
Number of patients	35		79	
Gender (male vs. Female)	62.9 % vs. 37.1 %	35	48.1 % vs. 51.9	79
Age at seizure onset (years)	12.5 ± 1.6	29	12.7 ± 1.6	70
Seizure frequency per month	27.8 ± 8.1	34	8.1 ± 1.0	79
Drug therapy (sodium-channel blocker monotherapy vs. LEV combinations vs. non LEV-combinations	17.1 % vs. 34.3 % vs. 48.6 %	35	20.3 % vs. 36.7 % vs. 43.0 %	79
Age at epilepsy surgery (years)	22.8 ± 2.7	35	35.0 ± 1.7	79
Post-operative outcome (Engel IA vs. Engel IV B)	55.9 % vs. 44.1 %	34	61.8 % vs. 38.2 %	76

(Parameters including gender, post-operative outcome and drug therapy are presented in percentage values. Age at seizure onset in years, age at epilepsy surgery in years, seizure frequency per month are presented in mean ± SEM values. The post-operative outcome is classified according Engel classification (class I A: completely seizure free; class IV B: no seizure reduction).

Supplementary Notes

Supplementary Note 1.

Reasoning for lesion-associated TLE patients as controls for HS-TLE in Ca_V3.2/MTF1 mRNA correlations: All patients included here had seizure activity prior to epilepsy surgery, i.e. as part of the definition of pharmacoresistant focal epilepsy. In the HS patient group, no individual was suffering from recurrent seizures at the time of birth but developed chronic recurrent seizures and HS later in life. For many patients of the present HS cohort, transient insults including prolonged febrile seizures during childhood could be recapitulated. In contrast, in 'lesion-associated' hippocampi, TLE is sufficiently explained by the tumor or malformation in the vicinity of the hippocampal formation. Respective patients lack clinical histories of initial precipitating events/epileptogenesis and the hippocampal formation does not reveal the damage pattern of HS. Therefore, we here used 'lesion-associated' TLE hippocampi as non-epileptogenic control for epileptogenic HS hippocampi.

Supplementary Note 2.

Representative epileptogenesis history of a TLE patient: The patient experienced a first time episode of non-convulsive status epilepticus at home and was referred to our University Hospital. An initial EEG during the “postictal” phase was diffusely slow with theta rhythmicity of about 6 Hz and individual abortive sharp-slow waves, which was interpreted as right fronto-temporo-central focus with signs of increased excitability. At the time of referral, MRI demonstrated hippocampal swelling associated with cytotoxic edema pronounced in the CA1 sector as revealed by coronal T2-weighted fast spin echo and axial diffusion-weighted spin echo EPI images. The differential diagnosis of imaging included changes after SE versus limbic/herpes encephalitis[1-3]. However, the clinical history as well as the lack of (a) specific autoantibodies (antibodies against amphiphysin, CV2, PNMA2 Ma-2/Ta, Ri, Yo, Hu, Recoverin, SOX1, Titin, GAD65, Zic, Tr; glutamate receptor type NMDA, type AMPA1, type AMPA2 as well as VGKC-autoantibodies against CASPR2 and LGI1, GABA B-receptor, GAD65) and (b) the absence of DNA/ autoantibodies for various potentially relevant viruses including FSME in cerebrospinal fluid argued strongly against the encephalitis pathogenesis. Within only two months after this initial event, cytotoxic edema disappeared and hippocampal atrophy, i.e. HS has manifested on MRI. Under levetiracetam up to 3000 mg daily, seizures could be controlled.

Supplementary References

1. Urbach, H.*, et al.* Serial MRI of limbic encephalitis. *Neuroradiology* **48**, 380-386 (2006).
2. Salmenpera, T., Kalviainen, R., Partanen, K. & Pitkanen, A. Hippocampal and amygdaloid damage in partial epilepsy: a cross-sectional MRI study of 241 patients. *Epilepsy research* **46**, 69-82 (2001).
3. Pohlmann-Eden, B., Gass, A., Peters, C.N., Wennberg, R. & Blumcke, I. Evolution of MRI changes and development of bilateral hippocampal sclerosis during long lasting generalised status epilepticus. *Journal of neurology, neurosurgery, and psychiatry* **75**, 898-900 (2004).
